# Supplementary material for: Automatic learning mechanisms for flexible human locomotion
Source: eLife. 2026 Feb 3;13:RP101671. doi: 10.7554/eLife.101671 (PMC12867481; doi:10.7554/eLife.101671)
Supplement: Supplementary file 1. [file elife-101671-supp1.docx]

**Automatic learning mechanisms
for flexible human locomotion**

**Cristina Rossi ^a,b^, Kristan A. Leech ^c,d^, Ryan T. Roemmich ^b,e^, Amy J. Bastian ^a,b,*^**

^a^ Department of Neuroscience, The Johns Hopkins University School of Medicine, Baltimore, MD, 21205, USA; ^b^ Center for Movement Studies, Kennedy Krieger Institute, Baltimore, MD, 21205, USA; ^c^ Division of Biokinesiology and Physical Therapy, University of Southern California, Los Angeles, CA, 90033, USA; ^d^ Neuroscience Graduate Program, University of Southern California, Los Angeles, CA, 90007, USA; ^e^ Department of Physical Medicine and Rehabilitation, The Johns Hopkins University School of Medicine, Baltimore, MD, 21205, USA. * Corresponding author, bastian@kennedykrieger.org.

**Supplementary file 1**

**Supplementary Tables with Statistical Results for Experiment 1 and Control Experiments**

**Supplementary file 1-table 1**

**Experiment 1, CI of step length asymmetry for each speed in the Ramp Up and Ramp Down tasks.** 95% bootstrapped confidence interval was corrected for multiple comparisons using false discovery rate, Ramp Up $\boldsymbol{\alpha}_{\mathbf{corrected}}\mathbf{=}\frac{\mathbf{6 significant comparisons}}{\mathbf{7 total comparisons}}\mathbf{*0.05=0.0429}$, Ramp Down $\boldsymbol{\alpha}_{\mathbf{corrected}}\mathbf{=}\frac{\mathbf{12 significant comparisons}}{\mathbf{21 total comparisons}}\mathbf{*0.05=0.0286}$. Corrected CIs significantly different from zero are highlighted. Left speed was constant at 0.5m/s.

|  | **right speed (m/s)** | **mean** | **95% CI** | **Corrected CI** |
| --- | --- | --- | --- | --- |
| **Baseline**  **Ramp Up** | 0.35 | 0.203 | [0.177, 0.231] | **{0.175, 0.232}** |
|  | 0.4 | 0.078 | [0.049, 0.105] | **{0.048, 0.106}** |
|  | 0.45 | 0.006 | [-0.017, 0.028] | - |
|  | 0.5 | -0.042 | [-0.064, -0.020] | **{-0.065, -0.019}** |
|  | 0.55 | -0.088 | [-0.126, -0.050] | **{-0.127, -0.049}** |
|  | 0.6 | -0.123 | [-0.149, -0.098] | **{-0.150, -0.097}** |
|  | 0.65 | -0.144 | [-0.171, -0.119] | **{-0.172, -0.118}** |
| **Post-adaptation**  **Ramp Down** | 1.5 | -0.015 | [-0.038, 0.009] | - |
|  | 1.45 | -0.011 | [-0.035, 0.014] | - |
|  | 1.4 | -0.01 | [-0.030, 0.010] | - |
|  | 1.35 | 0.002 | [-0.025, 0.029] | - |
|  | 1.3 | 0 | [-0.027, 0.031] | - |
|  | 1.25 | 0.015 | [-0.013, 0.045] | - |
|  | 1.2 | 0.018 | [-0.004, 0.042] | - |
|  | 1.15 | 0.005 | [-0.019, 0.029] | - |
|  | 1.1 | 0.024 | [0.000, 0.052] | {-0.002, 0.056} |
|  | 1.05 | 0.023 | [0.002, 0.044] | {-0.001, 0.047} |
|  | 1 | 0.026 | [-0.001, 0.052] | - |
|  | 0.95 | 0.038 | [0.014, 0.059] | **{0.011, 0.062}** |
|  | 0.9 | 0.056 | [0.026, 0.083] | **{0.023, 0.087}** |
|  | 0.85 | 0.067 | [0.036, 0.097] | **{0.032, 0.100}** |
|  | 0.8 | 0.083 | [0.056, 0.109] | **{0.053, 0.113}** |
|  | 0.75 | 0.112 | [0.084, 0.138] | **{0.081, 0.142}** |
|  | 0.7 | 0.126 | [0.091, 0.160] | **{0.087, 0.164}** |
|  | 0.65 | 0.154 | [0.117, 0.192] | **{0.113, 0.196}** |
|  | 0.6 | 0.174 | [0.143, 0.206] | **{0.139, 0.209}** |
|  | 0.55 | 0.204 | [0.166, 0.243] | **{0.162, 0.248}** |
|  | 0.5 | 0.213 | [0.172, 0.254] | **{0.168, 0.259}** |

**Supplementary file 1-table 2**

Control experiments, within-group difference between $\boldsymbol{compensatio}\boldsymbol{n}_{\text{motor total}}$ and $\boldsymbol{compensatio}\boldsymbol{n}_{\text{perceptual}}$. 95% bootstrapped confidence interval was corrected for multiple comparisons using false discovery rate, $\boldsymbol{\alpha}_{\mathbf{corrected}}\mathbf{=}\frac{\mathbf{7 significant comparisons}}{\mathbf{7 total comparisons}}\mathbf{*0.05=0.05}$. Significant differences based on corrected CI are highlighted.

| **Group** | **Mean** | **95% CI** | **Corrected CI** |
| --- | --- | --- | --- |
| Short Ascend | 47.450 | [36.082, 59.494] | **{36.082, 59.494}** |
| Medium Ascend | 48.295 | [38.795, 57.273] | **{38.795, 57.273}** |
| Long Ascend | 55.565 | [44.325, 66.551] | **{44.325, 66.551}** |
| Short Descend | 36.252 | [27.434, 44.096] | **{27.434, 44.096}** |
| Medium Descend | 56.012 | [42.462, 69.950] | **{42.462, 69.950}** |
| Small Abrupt | 36.224 | [17.254, 55.718] | **{17.254, 55.718}** |
| Small Gradual | 15.824 | [3.975, 27.015] | **{3.975, 27.015}** |

**Supplementary file 1-table 3**

**Control experiments, CI of step length asymmetry in the first and last stride of the speed match task** (first post-adaptation task). 95% bootstrapped confidence interval was corrected for multiple comparisons using false discovery rate, first stride $\boldsymbol{\alpha}_{\mathbf{corrected}}\mathbf{=}\frac{\mathbf{5 significant comparisons}}{\mathbf{7 total comparisons}}\mathbf{*0.05=0.0357}$, last stride $\boldsymbol{\alpha}_{\mathbf{corrected}}\mathbf{=}\frac{\mathbf{2 significant comparisons}}{\mathbf{7 total comparisons}}\mathbf{*0.05=0.0143}$. Corrected CIs significantly different from zero are highlighted.

|  | **Group** | **mean** | **95% CI** | **Corrected CI** |
| --- | --- | --- | --- | --- |
| **First stride** | ShortAscend | 0.4572 | [0.3554, 0.5612] | **{0.3474, 0.5685}** |
|  | MediumAscend | 0.433 | [0.2786, 0.5999] | **{0.2712, 0.6125}** |
|  | LongAscend | 0.3887 | [0.1106, 0.6414] | **{0.0909, 0.6569}** |
|  | ShortDescend | -0.1385 | [-0.1846, -0.1001] | **{-0.1880, -0.0972}** |
|  | MediumDescend | -0.0165 | [-0.0691, 0.0393] | - |
|  | SmallAbrupt | -0.0308 | [-0.0693, 0.0030] | - |
|  | SmallGradual | -0.0648 | [-0.1210, -0.0063] | **{-0.1240, -0.0023}** |
| **Last stride** | ShortAscend | -0.014 | [-0.0291, 0.0007] | - |
|  | MediumAscend | 0.0021 | [-0.0396, 0.0445] | - |
|  | LongAscend | 0.0224 | [0.0003, 0.0471] | {-0.0045, 0.0542} |
|  | ShortDescend | -0.0352 | [-0.0695, -0.0025] | {-0.0778, 0.0052} |
|  | MediumDescend | 0.0431 | [-0.0004, 0.0899] | - |
|  | SmallAbrupt | 0.0306 | [-0.0005, 0.0617] | - |
|  | SmallGradual | -0.0041 | [-0.0323, 0.0246] | - |

**Supplementary file 1-table 4**

**Control experiments, within-group comparison of** $\boldsymbol{compensatio}\boldsymbol{n}_{\text{motor total}}$ **to 100%.** 95% bootstrapped confidence interval was corrected for multiple comparisons using false discovery rate, $\alpha_{corrected}=\frac{5 significant comparisons}{7 total comparisons}*0.05=0.0357$. Corrected CIs significantly different from 100% are highlighted.

| **Group** | **Mean** | **95% CI** | **Corrected CI** |
| --- | --- | --- | --- |
| ShortAscend | 73.200 | [65.330, 81.111] | **{64.812, 81.563}** |
| MediumAscend | 90.045 | [86.579, 93.965] | **{86.336, 94.239}** |
| LongAscend | 98.815 | [93.457, 104.583] | - |
| ShortDescend | 70.752 | [63.513, 77.437] | **{63.013, 77.843}** |
| MediumDescend | 91.012 | [84.132, 98.274] | **{83.758, 98.773}** |
| SmallAbrupt | 98.349 | [86.146, 111.269] | - |
| SmallGradual | 73.074 | [61.812, 85.213] | **{61.141, 85.994}** |

**Supplementary file 1-table 5**

**Control experiments, between-group differences in** $\boldsymbol{compensatio}\boldsymbol{n}_{\text{motor total}}$**.** 95% bootstrapped confidence interval was corrected for multiple comparisons using false discovery rate, $\alpha_{corrected}=\frac{3 significant comparisons}{6 total comparisons}*0.05=0.025$. Significant differences based on corrected CI are highlighted.

| **Groups** | **Mean** | **95% CI** | **Corrected CI** |
| --- | --- | --- | --- |
| MediumAscend - ShortAscend | 16.845 | [7.981, 25.580] | **{7.029, 26.780}** |
| LongAscend - MediumAscend | 8.770 | [2.197, 15.529] | **{1.126, 16.418}** |
| ShortDescend - ShortAscend | -2.448 | [-13.194, 7.983] | - |
| MediumDescend - MediumAscend | 0.967 | [-6.939, 9.079] | - |
| SmallAbrupt - MediumDescend | 7.336 | [-6.881, 21.821] | - |
| SmallGradual - SmallAbrupt | -25.275 | [-42.137, -7.837] | **{-44.829, -5.784}** |

**Supplementary file 1-table 6**

**Control experiments, between-group differences in recalibration contribution to total output** ($compensation_{\text{perceptual}}$ / $compensation_{\text{motor total}}$). 95% bootstrapped confidence interval was corrected for multiple comparisons using false discovery rate, $\alpha_{corrected}=\frac{1 significant comparison}{6 total comparisons}*0.05=0.0083$. Significant differences based on corrected CI are highlighted.

| **Groups** | **Mean** | **95% CI** | **Corrected CI** |
| --- | --- | --- | --- |
| Medium Ascend – Short Ascend | 9.277 | [-5.880, 24.373] | - |
| Long Ascend – Medium Ascend | -2.049 | [-17.108, 12.192] | - |
| Short Descend – Short Ascend | 12.343 | [-3.167, 28.054] | - |
| Medium Descend – Medium Ascend | -7.303 | [-24.239, 9.448] | - |
| Small Abrupt – Medium Descend | 26.546 | [7.235, 47.436] | **{0.488, 54.744}** |
| Small Gradual – Small Abrupt | 16.238 | [-5.608, 38.497] | - |

**Supplementary file 1-table 7**

**Control experiments, within-group difference between** $\boldsymbol{compensatio}\boldsymbol{n}_{\text{motor recalibration}}$ **and** $\boldsymbol{compensatio}\boldsymbol{n}_{\text{perceptual}}$ **across washout.** 95% bootstrapped confidence interval was corrected for multiple comparisons using false discovery rate. $\boldsymbol{\alpha}_{\mathbf{corrected}}\mathbf{=}\frac{\mathbf{3 significant comparisons}}{\mathbf{6 total comparisons}}\mathbf{*0.05=0.0250}$ for Short Ascend; $\boldsymbol{\alpha}_{\mathbf{corrected}}\mathbf{=}\frac{\mathbf{5 significant comparisons}}{\mathbf{6 total comparisons}}\mathbf{*0.05=0.0417}$ for Medium and Long Ascend. Significant differences based on corrected CI are highlighted.

|  | **Time post-adaptation (min)** | **Mean** | **95% CI** | **Corrected CI** |
| --- | --- | --- | --- | --- |
| **Short Ascend** | 0 | -0.074 | [-0.238, 0.076] | - |
|  | 1 | -0.173 | [-0.280, -0.074] | **{-0.296, -0.062}** |
|  | 2 | -0.113 | [-0.197, -0.032] | **{-0.210, -0.022}** |
|  | 4 | -0.074 | [-0.117, -0.033] | **{-0.123, -0.028}** |
|  | 8 | -0.045 | [-0.094, 0.004] | - |
|  | 16 | -0.038 | [-0.127, 0.060] | - |
| **Medium Ascend** | 0 | -0.033 | [-0.249, 0.209] | - |
|  | 1 | -0.183 | [-0.379, -0.001] | {-0.384, 0.005} |
|  | 2 | -0.231 | [-0.381, -0.067] | **{-0.387, -0.061}** |
|  | 4 | -0.111 | [-0.190, -0.029] | **{-0.193, -0.027}** |
|  | 8 | -0.216 | [-0.344, -0.094] | **{-0.349, -0.089}** |
|  | 16 | -0.163 | [-0.255, -0.059] | **{-0.258, -0.055}** |
| **Long Ascend** | 0 | 0.218 | [-0.018, 0.456] | - |
|  | 1 | -0.209 | [-0.374, -0.051] | **{-0.380, -0.044}** |
|  | 2 | -0.14 | [-0.260, -0.034] | **{-0.265, -0.031}** |
|  | 4 | -0.11 | [-0.195, -0.024] | **{-0.198, -0.021}** |
|  | 8 | -0.126 | [-0.239, -0.017] | **{-0.243, -0.013}** |
|  | 16 | -0.113 | [-0.213, -0.027] | **{-0.218, -0.025}** |
