# Supplementary material for: Automatic learning mechanisms for flexible human locomotion
Source: eLife. 2026 Feb 3;13:RP101671. doi: 10.7554/eLife.101671 (PMC12867481; doi:10.7554/eLife.101671)
Supplement: Supplementary file 2. [file elife-101671-supp2.docx]

**Automatic learning mechanisms
for flexible human locomotion**

**Cristina Rossi ^a,b^, Kristan A. Leech ^c,d^, Ryan T. Roemmich ^b,e^, Amy J. Bastian ^a,b,*^**

^a^ Department of Neuroscience, The Johns Hopkins University School of Medicine, Baltimore, MD, 21205, USA; ^b^ Center for Movement Studies, Kennedy Krieger Institute, Baltimore, MD, 21205, USA; ^c^ Division of Biokinesiology and Physical Therapy, University of Southern California, Los Angeles, CA, 90033, USA; ^d^ Neuroscience Graduate Program, University of Southern California, Los Angeles, CA, 90007, USA; ^e^ Department of Physical Medicine and Rehabilitation, The Johns Hopkins University School of Medicine, Baltimore, MD, 21205, USA. * Corresponding author, bastian@kennedykrieger.org.

**Supplementary file 2**

**Supplementary Tables with Statistical Results for Experiment 2 and Clustering Analysis**

**Supplementary file 2-table 1**

**Experiment 2, clustering analysis measures and results for individual participants.** Left column: 95% confidence interval of each participant’s baseline (second tied-belt block). Middle column: number of strides in the first portion of the Ramp Up & Down (right speeds larger than adaptation) with step length asymmetry below the participant’s own baseline CI. Right column: clustering classification (cluster 1, memory; or cluster 2, structure).

| **Participant** | **BL mean [95% CI]** | **# strides < CI** | **Cluster** |
| --- | --- | --- | --- |
| P1 | 0.027 [0.016, 0.039] | 21 | Cluster 2 (structure) |
| P2 | 0.009 [0.004, 0.015] | 42 | Cluster 1 (memory) |
| P3 | -0.007 [-0.014, -0.000] | 55 | Cluster 1 (memory) |
| P4 | -0.017 [-0.025, -0.008] | 14 | Cluster 2 (structure) |
| P5 | 0.094 [0.082, 0.107] | 58 | Cluster 1 (memory) |
| P6 | 0.072 [0.060, 0.084] | 50 | Cluster 1 (memory) |
| P7 | 0.004 [-0.002, 0.011] | 21 | Cluster 2 (structure) |
| P8 | 0.024 [0.015, 0.032] | 51 | Cluster 1 (memory) |
| P9 | -0.045 [-0.053, -0.037] | 44 | Cluster 1 (memory) |
| P10 | -0.020 [-0.028, -0.013] | 3 | Cluster 2 (structure) |
| P11 | 0.002 [-0.007, 0.010] | 60 | Cluster 1 (memory) |
| P12 | -0.043 [-0.055, -0.032] | 16 | Cluster 2 (structure) |
| P13 | -0.027 [-0.037, -0.016] | 40 | Cluster 1 (memory) |
| P14 | 0.007 [-0.009, 0.024] | 58 | Cluster 1 (memory) |
| P15 | -0.055 [-0.068, -0.043] | 4 | Cluster 2 (structure) |
| P16 | -0.008 [-0.018, 0.003] | 40 | Cluster 1 (memory) |
| P17 | -0.068 [-0.081, -0.055] | 16 | Cluster 2 (structure) |
| P18 | -0.034 [-0.042, -0.026] | 11 | Cluster 2 (structure) |
| P19 | -0.009 [-0.017, -0.000] | 56 | Cluster 1 (memory) |
| P20 | 0.008 [-0.007, 0.022] | 38 | Cluster 1 (memory) |

**Supplementary file 2-table 2**

**Experiment 2, CI of step length asymmetry for each speed in the Ramp Up and magenta Ramp Up & Down tasks.** 95% bootstrapped confidence interval was corrected for multiple comparisons using false discovery rate, Ramp Up $\boldsymbol{\alpha}_{\mathbf{corrected}}\mathbf{=}\frac{\mathbf{6 significant comparisons}}{\mathbf{7 total comparisons}}\mathbf{*0.05=0.0429}$, magenta Ramp Up & Down $\boldsymbol{\alpha}_{\mathbf{corrected}}\mathbf{=}\frac{\mathbf{17 significant comparisons}}{\mathbf{21 total comparisons}}\mathbf{*0.05=0.0405}$. Corrected CIs significantly different from zero are highlighted. Left speed was constant at 0.5m/s.

|  | **right speed (m/s)** | **mean** | **95% CI** | **Corrected CI** |
| --- | --- | --- | --- | --- |
| **Baseline**  **Ramp Up** | 0.35 | 0.197 | [0.145, 0.260] | **{0.143, 0.262}** |
|  | 0.4 | 0.054 | [0.025, 0.081] | **{0.023, 0.082}** |
|  | 0.45 | 0.015 | [-0.017, 0.048] | - |
|  | 0.5 | -0.039 | [-0.066, -0.010] | **{-0.067, -0.009}** |
|  | 0.55 | -0.068 | [-0.090, -0.045] | **{-0.090, -0.044}** |
|  | 0.6 | -0.096 | [-0.118, -0.076] | **{-0.119, -0.075}** |
|  | 0.65 | -0.109 | [-0.131, -0.089] | **{-0.132, -0.088}** |
| **Post-adaptation magenta Ramp Up & Down** | 1.5 | 0.008 | [-0.009, 0.026] | - |
|  | 1.45 | 0 | [-0.016, 0.018] | - |
|  | 1.4 | 0.018 | [-0.001, 0.040] | - |
|  | 1.35 | 0.03 | [0.007, 0.056] | **{0.006, 0.057}** |
|  | 1.3 | 0.028 | [0.003, 0.054] | **{0.002, 0.055}** |
|  | 1.25 | 0.038 | [0.015, 0.062] | **{0.014, 0.064}** |
|  | 1.2 | 0.041 | [0.019, 0.063] | **{0.018, 0.064}** |
|  | 1.15 | 0.029 | [-0.001, 0.054] | - |
|  | 1.1 | 0.043 | [0.017, 0.074] | **{0.016, 0.076}** |
|  | 1.05 | 0.043 | [0.016, 0.069] | **{0.014, 0.071}** |
|  | 1 | 0.051 | [0.019, 0.086] | **{0.018, 0.087}** |
|  | 0.95 | 0.068 | [0.038, 0.101] | **{0.037, 0.102}** |
|  | 0.9 | 0.072 | [0.048, 0.098] | **{0.047, 0.100}** |
|  | 0.85 | 0.074 | [0.049, 0.099] | **{0.048, 0.101}** |
|  | 0.8 | 0.083 | [0.058, 0.113] | **{0.057, 0.114}** |
|  | 0.75 | 0.096 | [0.068, 0.126] | **{0.067, 0.127}** |
|  | 0.7 | 0.115 | [0.085, 0.146] | **{0.084, 0.148}** |
|  | 0.65 | 0.135 | [0.101, 0.173] | **{0.100, 0.176}** |
|  | 0.6 | 0.188 | [0.134, 0.251] | **{0.132, 0.254}** |
|  | 0.55 | 0.174 | [0.131, 0.222] | **{0.129, 0.224}** |
|  | 0.5 | 0.233 | [0.163, 0.313] | **{0.160, 0.318}** |

**Supplementary file 2-table 3**

**Experiment 1, clustering analysis measures and results for individual participants.** Left column: 95% confidence interval of each participant’s baseline (second tied-belt block). Middle column: number of strides in the ramp down with step length asymmetry above the participant’s own baseline CI. Right column: clustering classification (cluster 1 or outlier).

| **Participant** | **BL mean [95% CI]** | **# strides > CI** | **Cluster** |
| --- | --- | --- | --- |
| P1 | 0.013 [0.005, 0.021] | 38 | Cluster 1 |
| *P2* | *-0.002 [-0.009, 0.006]* | *16* | *outlier* |
| *P3* | *0.106 [0.092, 0.121]* | *13* | *outlier* |
| P4 | 0.005 [-0.008, 0.018] | 53 | Cluster 1 |
| P5 | 0.019 [0.007, 0.032] | 47 | Cluster 1 |
| P6 | -0.040 [-0.058, -0.024] | 57 | Cluster 1 |
| P7 | -0.009 [-0.019, 0.002] | 58 | Cluster 1 |
| P8 | 0.027 [0.017, 0.037] | 25 | Cluster 1 |
| P9 | -0.019 [-0.030, -0.009] | 37 | Cluster 1 |
| P10 | -0.007 [-0.016, 0.003] | 48 | Cluster 1 |
| P11 | -0.038 [-0.045, -0.032] | 29 | Cluster 1 |
| P12 | -0.012 [-0.025, 0.000] | 60 | Cluster 1 |
| P13 | 0.022 [0.013, 0.030] | 40 | Cluster 1 |
| P14 | -0.046 [-0.054, -0.039] | 40 | Cluster 1 |
| P15 | -0.001 [-0.010, 0.008] | 33 | Cluster 1 |
| P16 | -0.066 [-0.073, -0.059] | 47 | Cluster 1 |
| P17 | 0.027 [0.016, 0.038] | 59 | Cluster 1 |
| P18 | 0.011 [-0.001, 0.024] | 38 | Cluster 1 |
| P19 | -0.060 [-0.077, -0.043] | 49 | Cluster 1 |
| P20 | 0.048 [0.039, 0.057] | 30 | Cluster 1 |

**Supplementary file 2-table 4**

**Experiment 1, replication of statistical analyses after removal of the 2 outliers detected with the primary clustering analysis.** Measures are reported the same way as the original analysis (rows 1-7 report mean [95% CI] {corrected CI}; rows 8-9 report correlation coefficient and p value). Significant results are highlighted. Removing the outliers did not affect the statistical significance of any of the tests. UB = upper bound, LB = lower bound.

| **Measure** | **Statistical Results** |
| --- | --- |
| “Recalibration Only” (dual state) $-$ “Recalibration + Mapping” BIC | 8.435 [3.302, 13.896] **{3.302, 13.896}** |
| “Memory of Errors” $-$ “Recalibration + Mapping” BIC | 11.522 [5.769, 17.791] **{5.769, 17.791}** |
| “Optimal Control” $-$ “Recalibration + Mapping” BIC | 19.125 [13.956, 24.512] **{13.956, 24.512}** |
| $compensation_{\text{motor total}}-compensation_{\text{perceptual UB}}$ | 30.574 [23.788, 37.855] **{23.788, 37.855}** |
| $compensation_{\text{motor total}}-compensation_{\text{perceptual LB}}$ | 58.074 [49.016, 67.602] **{49.016, 67.602}** |
| $compensation_{\text{motor recalibration}}- compensation_{\text{perceptual UB}}$ | -7.606 [-14.425, -1.198] **{-14.425, -1.198}** |
| $compensation_{\text{motor recalibration}}- compensation_{\text{perceptual LB}}$ | 19.894 [9.259, 30.553] **{9.259, 30.553}** |
| Correlation between  $compensation_{\text{motor recalibration}}$ & $compensation_{\text{perceptual UB}}$ | r=0.59, **p=0.010** |
| Correlation between  $compensation_{\text{motor recalibration}}$ & $compensation_{\text{perceptual LB}}$ | r=0.30, p=0.226 |
