# Supplementary material for: Automatic learning mechanisms for flexible human locomotion
Source: eLife. 2026 Feb 3;13:RP101671. doi: 10.7554/eLife.101671 (PMC12867481; doi:10.7554/eLife.101671)
Supplement: Supplementary file 3. [file elife-101671-supp3.docx]

**Automatic learning mechanisms
for flexible human locomotion**

**Cristina Rossi ^a,b^, Kristan A. Leech ^c,d^, Ryan T. Roemmich ^b,e^, Amy J. Bastian ^a,b,*^**

^a^ Department of Neuroscience, The Johns Hopkins University School of Medicine, Baltimore, MD, 21205, USA; ^b^ Center for Movement Studies, Kennedy Krieger Institute, Baltimore, MD, 21205, USA; ^c^ Division of Biokinesiology and Physical Therapy, University of Southern California, Los Angeles, CA, 90033, USA; ^d^ Neuroscience Graduate Program, University of Southern California, Los Angeles, CA, 90007, USA; ^e^ Department of Physical Medicine and Rehabilitation, The Johns Hopkins University School of Medicine, Baltimore, MD, 21205, USA. * Corresponding author, bastian@kennedykrieger.org.

**Supplementary file 3**

**Questionnaire Responses for Experiment 2**

Experiment 2, responses to questionnaire. Raw responses of individual participants (center column) sorted by subgroup (left column), and classification of responses (three rightmost columns) used for Fig. 10.

| ***Response to:***  Did you deliberately change how you walked to account for how fast the belts were moving? If so, describe how.  *Note:* *deliberately means that you thought about and decided to move that way* | | | **deliberate?** | **relevant?** | **accurate?** |
| --- | --- | --- | --- | --- | --- |
| **Memory-Based** | P2 | I noticed that I was making heavy steps on the right foot which caused shifting in my upper body so I tried to control that a little bit, but not much | Y | N | - |
|  | P3 | Yes, I tried to adjust how fast I changed foot while walking, and tried to shift more weight onto my left side which was walking at a slower pace. | Y | Y | N |
|  | P5 | Yes, I need to change how I walk because the belt moves in different speed | Y | N | - |
|  | P6 | Yes, I changed my pace to stay upright and not fall down | Y | N | - |
|  | P8 | No | N | - | - |
|  | P9 | Yes, I started to change how I walked when I realized the right belt was moving faster, and I had to compensate on my left to stabilize my body. | Y | N | - |
|  | P11 | No. | N | - | - |
|  | P13 | Yes. I tried to get into a rhythm so that I had consistently long right strides and short left strides. | Y | N | - |
|  | P14 | Yes, I tried to bow out my right leg(for the period of the time where the right belt was moving faster) so as to give me additional balance. I also tried to sync up my right foot moving up off the track at the same or close time as my left foot hit the track for the portion of the block where the right track was moving faster | Y | Y | N |
|  | P20 | Yes, as soon as I felt the right belt's speed increase, I tried to spend as much time leaning on my left leg as possible and wait for a little before switching to my right foot (and then spending less time on my right foot) | Y | Y | Y |
| **Structure-Based** | P1 | Matched duration of standing on each foot by moving the right leg faster than the left leg and at a wider angle (range of motion). | Y | Y | N |
|  | P4 | YES, I put less pressure on my right leg so I wouldn't lose my balance as the belts were moving at different rates. | Y | N | - |
|  | P7 | Tried to even out the limp caused by the different leg speeds | Y | Y | N |
|  | P10 | Yes I deliberately put more weight on my left foot (on the belt that was slower) to provide more stability for my right foot that was moving faster. | Y | N | - |
|  | P12 | I felt like I had to adjust my speed to keep up with the way the belts were moving differently, I was thinking about how the right belt was moving faster but I didn't really feel like I had control over my own gait | N | - | - |
|  | P15 | Yes, I adjusted as if I was limping. | Y | Y | N |
