# Supplementary material for: Automatic learning mechanisms for flexible human locomotion
Source: eLife. 2026 Feb 3;13:RP101671. doi: 10.7554/eLife.101671 (PMC12867481; doi:10.7554/eLife.101671)
Supplement: Supplementary file 4. [file elife-101671-supp4.docx]

**Automatic learning mechanisms
for flexible human locomotion**

**Cristina Rossi ^a,b^, Kristan A. Leech ^c,d^, Ryan T. Roemmich ^b,e^, Amy J. Bastian ^a,b,*^**

^a^ Department of Neuroscience, The Johns Hopkins University School of Medicine, Baltimore, MD, 21205, USA; ^b^ Center for Movement Studies, Kennedy Krieger Institute, Baltimore, MD, 21205, USA; ^c^ Division of Biokinesiology and Physical Therapy, University of Southern California, Los Angeles, CA, 90033, USA; ^d^ Neuroscience Graduate Program, University of Southern California, Los Angeles, CA, 90007, USA; ^e^ Department of Physical Medicine and Rehabilitation, The Johns Hopkins University School of Medicine, Baltimore, MD, 21205, USA. * Corresponding author, bastian@kennedykrieger.org.

**Supplementary file 4**

**Pseudocode for the clustering analysis**

**Pseudocode for the clustering analysis**

*Notation (A x B) indicates matrix size. N = 20, K = 20 (i.e. the number of participants)*

1. Initialize BestEpsilon (1 x K), BestMinPts (1 x K), BestNumberOutliers (1 x K), BestNumberClusters (1 x K)
2. Repeats steps 1-12 for k=1:K:
3. Dst (N x N) = distance of between each pair of participants:

for n1=1:N

for n2=1:N

Dst (n1,n2) = abs (measure(n1) – measure(n2) )

1. AvgDst (N x 1) = mean distance to the first ‘k’ neighbors (for each participant):

for n=1:N

AvgDst(n) = mean ( Dst(n,1:k) )

1. SortAvgDst (N x 1) = AvgDst sorted in ascending order
2. SortAvgDstD (N-1 x 1) = first derivative of SortAvgDst
3. SortAvgDstDD (N-2 x 1) = second derivative of SortAvgDst
4. iEpsilon (M x 1) = indexes i where SortAvgDstDD (i) > 1/100 * mean(SortAvgDstD)

*(size M is automatic based on step 6)*

1. Epsilon (M x 1) = SortAvgDst(iEpsilon+1)
2. SortEpsilon (M x 1) = Epsilon (j) where SortAvgDstDD (j) is sorted in descending order (i.e. sort Epsilon by descending SortAvgDstDD values)
3. MinPts (M x 1):

for m=1:M

initialize NumberNeighbors (N x 1)

for n=1:N

NumberNeighbors(n) = number of elements for which Dst (n, : ) ≤ SortEpsilon(m)

MinPts(m) = mean (NumberNeighbors)

1. Eliminate SortEpsilon (j) and MinPts (j) for which MinPts (j) < 2 or MinPts (j) > 10
2. NumberOutliers (Q x 1): *(Q is the size of SortEpsilon and MinPts after step 10)*

for q=1:Q

clusters = dbscan (measure, SortEpsilon (q), MinPts(q) )

NumberOutliers(q) = number of participants not assigned to any clusters

*(e.g. NumberOutliers(q) = number of elements for which clusters == -1,*

*with -1 = the label for outliers)*

NumberClusters(q) = number of different clusters

*(e.g. NumberCluster(q) = number of unique clusters different than -1,*

*with -1 = the label for outliers)*

1. BestEpsilon(k), BestMinPts(k), BestNumberOutliers(k), BestNumberClusters(k) = SortEpsilon(q), MinPts(q), NumberOutliers(q), NumberClusters(q), where q is the smallest value for which NumberOutliers(q) == min(NumberOutliers)
2. OutlierBestEpsilon (J x 1), OutlierBestMinPts (J x 1), OutlierBestNumberClusters (J x 1) = BestEpsilon(j), BestMinPts(j), BestNumberClusters(j) for all j such that BestNumberOutliers(j) == min(BestNumberOutliers) *(size J is automatic based on step 12)*
3. OverallBestEspilon (1 x 1), OverallBestMinPts (1 x 1) = OutlierBestEpsilon(j), OutlierBestMinPts(j) for j such that OutlierBestNumberClusters(j) == min(OutlierBestNumberClusters)

*(note that in our dataset, we could choose any j in the last step and the resulting clustering is the same. This may not be true for different datasets)*
